# Effect of diode laser irradiation (780–980 nm) on resin–dentin microtensile bond strength: a systematic review of *in vitro* studies

**DOI:** 10.3389/fdmed.2026.1857302

**Published:** 2026-07-22

**Authors:** Chandan R K, Darshana Devadiga, Teena Sheethal Dsouza, Astrid Ana Gomes, Medhini R Bhagwat

**Affiliations:** Department of Conservative Dentistry and Endodontics, AB Shetty Memorial Institute of Dental Sciences (ABSMIDS), Nitte (Deemed to be University), Mangalore, India

**Keywords:** adhesive dentistry, dentin bonding, diode laser, *in vitro*, laser irradiation, microtensile bond strength, resin-dentin interface, systematic review

## Abstract

**Background:**

The intricate structural makeup of dentin and the hybrid layer's vulnerability to deterioration make it difficult to achieve long-lasting resin-dentin bonding. One technique for dentin biomodification that may enhance adhesive performance is diode laser irradiation.

**Objective:**

This systematic review aimed to evaluate the effect of diode laser irradiation (780–980 nm) on the microtensile bond strength (µTBS) of resin-dentin interfaces across *in vitro* studies.

**Methods:**

Following PRISMA 2020 guidelines and an OSF-registered protocol (OSF Registration ID: QJ6S8), an electronic search was conducted in PubMed, Scopus and Web of science for studies published between 2015 and 2025. *In vitro* studies using diode lasers (780–980 nm) on human dentin with µTBS outcomes were included. Risk of bias was assessed using the Quality Assessment Tool for *in vitro* studies tool.

**Results:**

Five *in vitro* studies met the inclusion criteria. Three studies reported statistically significant improvements in µTBS following diode laser pretreatment (range: approximately 30%–100% increase over controls), attributed to laser-induced collagen modification, reduced dentinal permeability, and enhanced resin infiltration. Two studies reported no statistically significant benefit, with suboptimal irradiation parameters identified as the likely contributing factor. Heterogeneity in irradiation protocols precluded meta-analysis.

**Conclusion:**

Diode laser irradiation at optimised parameters (940–970 nm, ≥1 W continuous mode) demonstrates a tendency to improve resin-dentin µTBS *in vitro*. However, the limited number of eligible studies, parameter heterogeneity, and absence of long-term clinical evidence preclude definitive clinical recommendations. Standardised protocols and controlled clinical trials are warranted.

**Systematic Review Registration:**

https://doi.org/10.17605/OSF.IO/QJ6S8, QJ6S8.

## Introduction

Bonding to dentin continues to present significant challenges in adhesive dentistry due to the complex structural, biological, and compositional characteristics. Unlike enamel, dentin contains a higher proportion of water and organic matrix, exhibits tubular fluid movement and microstructural variability, all of which influence resin infiltration and hybrid layer formation (1). These factors make the resin-dentin interface prone to hydrolytic degradation, incomplete polymerization, and mechanical instability over time (2). Furthermore, during bonding, the acid-etching process exposes a collagen network that is highly susceptible to degradation by endogenous proteolytic enzymes such as matrix metalloproteinases (MMPs) and cysteine cathepsins, which contribute to the gradual breakdown of the hybrid layer and reduction in long-term bond strength (3).

To address these inherent limitations, various biomodification approaches have been explored to enhance hybrid layer integrity, including the use of chemical cross-linkers, MMP inhibitors, biomimetic remineralization agents, and ethanol-wet bonding strategies (4). More recently, the application of lasers for dentin surface modification has gained attention as a potential method to improve the quality and longevity of adhesive interfaces. Lasers can induce morphological and physicochemical alterations on dentin, such as smear layer modification, reduction in permeability, and collagen reorganization, which may create more favorable conditions for adhesive infiltration and hybrid layer stability (5, 16, 17).

Among various laser systems, diode lasers operating at wavelengths between 780 and 980 nm have emerged as a practical and clinically applicable option. Their compact design, fibre-optic delivery, affordability, and ability to interact effectively with organic components make them suitable for intraoral applications. Diode laser irradiation produces localized thermal effects that may denature or shrink collagen fibrils, modify the smear layer, and increase dentin surface energy, thereby improving adhesive adaptation (6). These thermal changes may also reduce dentinal fluid flow and enhance the penetration of adhesive monomers into the underlying substrate, potentially increasing microtensile bond strength (µTBS) (7).

Although several *in vitro* studies have investigated the effect of diode laser pretreatment on resin-dentin bond strength, published findings have been inconsistent. Variability in laser wavelength, power settings, irradiation timing, and adhesive protocol has produced heterogeneous outcomes across studies, making it difficult to draw definitive conclusions from individual reports alone.

In light of the heterogeneous findings reported in the literature, a systematic evaluation of the available *in-vitro* evidence is essential to determine whether diode laser pretreatment provides a consistent bonding advantage and to identify the factors influencing its effectiveness.

Despite growing interest in laser-assisted dentin surface modification, no systematic review has specifically examined the effect of diode lasers within the 780–980 nm range on µTBS outcomes using a standardised quality appraisal framework for *in vitro* studies. It was hypothesised that diode laser pretreatment at optimised irradiation parameters would significantly enhance µTBS compared with untreated controls by inducing favourable photothermal modifications of the dentin substrate (16).

## Materials and methods

This systematic review was conducted in accordance with the PRISMA 2020 guidelines and was preregistered in the Open Science Framework(OSF ID: QJ6S8). Registration DOI 10.17605/OSF.IO/QJ6S8.

A comprehensive electronic search was performed in PubMed, Scopus, Web of Science and Cochrane library limited to studies published in the English language, as detailed in Table 1. Studies published between 2015 and 2025 were considered. The search period was restricted to 2015–2025 because preliminary scoping searches indicated that most studies investigating contemporary diode laser parameters (780–980 nm) and current adhesive systems were published during this period. Earlier studies predominantly evaluated outdated adhesive systems and laser protocols with limited clinical relevance. The search strategy involved combinations of keywords such as dentin, diode laser, laser irradiation, and microtensile bond strength using appropriate Boolean operators. Backward citation tracking identified numerous additional records; however, none met the predefined inclusion criteria and therefore no additional studies were included.

**Table 1:** Search strategy.

Database	Search strategy
PubMed	((dentin[Title/Abstract] OR dentine[Title/Abstract]) AND (“diode laser”[Title/Abstract] OR “laser diode”[Title/Abstract]) AND (“microtensile bond strength”[Title/Abstract] OR “bond strength”[Title/Abstract])) AND (“2015/01/01”[Date - Publication]: “2025/12/31”[Date - Publication])
Scopus	TITLE-ABS-KEY ((dentin OR dentine) AND (“diode laser” OR “laser diode” OR “semiconductor laser”) AND (“microtensile bond strength” OR “bond strength”) AND (“*in vitro*”)) AND PUBYEAR > 2014 AND PUBYEAR < 2027 AND PUBYEAR > 2014 AND PUBYEAR < 2027 AND PUBYEAR > 2014 AND PUBYEAR < 2027 AND (LIMIT-TO (SUBJAREA, “MEDI”)) AND (LIMIT-TO (LANGUAGE, “English”))
Web of science	*ALL=((dentin OR dentine) AND (“diode laser” OR “semiconductor laser” OR “laser irradiation”) AND (“microtensile bond strength” OR “bond strength”)) AND PY* *=* *(2015–2025)* “TS = (dentin OR dentine) AND TS = (‘diode laser’ OR ‘semiconductor laser’) AND TS = (‘microtensile bond strength’ OR ‘bond strength’)”
Cochrane library	(dentin OR dentine) AND (“diode laser” OR “laser diode” OR “semiconductor laser” OR “diode laser irradiation” OR “laser irradiation”) AND (“microtensile bond strength” OR “bond strength” OR “resin-dentin bond strength” OR µTBS)

The eligibility criteria for this review were defined according to the PICO framework. The population (P) included extracted human permanent teeth with dentin substrates. The intervention (I) consisted of diode laser irradiation within the wavelength range of 780–980 nm applied prior to adhesive procedures. The comparator (C) involved conventional bonding protocols performed without laser pretreatment. The outcome (O) assessed was microtensile bond strength (µTBS), expressed in megapascals (MPa). Only *in vitro* studies published in the English language between 2015 and 2025 were considered for inclusion.

A structured search was performed in PubMed, Scopus, Web of Science and Cochrane library as detailed in (Table 1).

Studies meeting the following inclusion criteria were selected: *in vitro* studies conducted on human dentin, studies evaluating diode lasers within the wavelength range of 780–980 nm, studies reporting microtensile bond strength outcomes, and articles published in English between 2015 and 2025. Exclusion criteria included non-English publications, *in vivo* studies, case reports, review articles, studies using non-diode laser systems, and studies not reporting microtensile bond strength outcomes.

**Figure 1:**
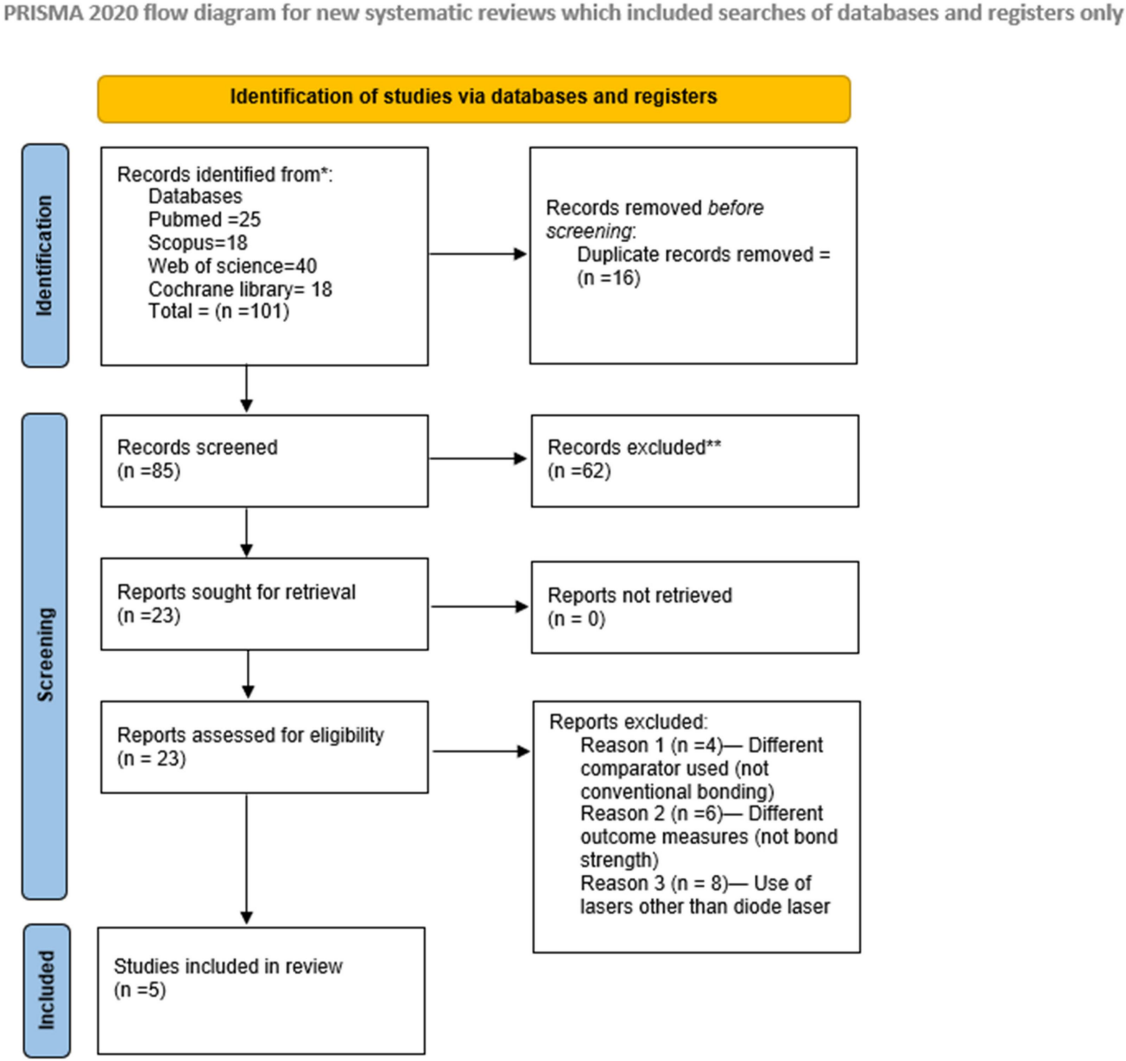
PRISMA flowchart.

The selection process involved initial screening of titles and abstracts, followed by full-text assessment of potentially eligible studies based on predefined criteria. The study selection process is illustrated in Figure 1.

*A priori* power calculations were not performed because the study design was a systematic review and did not involve recruitment of experimental samples.

### Quantitative synthesis

A quantitative meta-analysis was considered during protocol development. However, statistical pooling was deemed inappropriate because of substantial clinical and methodological heterogeneity among the included studies. Variations were observed in laser wavelength (810–970 nm), power settings (0.8–1 W), irradiation modes (continuous or pulsed), timing of irradiation relative to adhesive application, adhesive systems employed, aging procedures, and reporting of microtensile bond strength (µTBS) outcomes. Given the limited number of eligible studies (*n* = 5) and the anticipated high between-study heterogeneity, pooled effect estimates would likely be unreliable and potentially misleading. Therefore, a structured narrative synthesis was considered the most appropriate method for summarizing the available evidence.

However, to assess the sufficiency of the evidence base, the minimum number of studies required for a meaningful narrative synthesis was set at three independently conducted *in vitro* studies evaluating the same primary outcome (µTBS) with comparable interventions (diode laser irradiation within 780–980 nm). This threshold was met by the five eligible studies identified. The Grading of Recommendations Assessment, Development and Evaluation (GRADE) framework was not applied owing to the *in vitro* nature of all included studies, but the authors acknowledge this as a limitation.

### Study selection and data extraction

Two independent reviewers screened the titles, abstracts, and full texts of the retrieved studies using the Rayyan platform. Inter-reviewer agreement for study eligibility was assessed using Cohen's kappa statistic. Agreement was substantial at the title/abstract screening stage (*κ* = 0.85) and almost perfect at the full-text screening stage (*κ* = 0.92). The screening process was performed in a blinded manner, and any disagreements were resolved through discussion or consultation with a third reviewer. Data extraction was carried out using a standardized form, including study characteristics (author and year), laser parameters (wavelength, power, mode, application time, energy), dentin substrate, adhesive system, microtensile bond strength values for both laser-treated and control groups, aging conditions, and overall study outcomes, as presented in Table 2.

**Table 2:** Data extraction table.

Study	Wavelength (nm)	Power (W)	Mode	Application time (s)	Energy (J)	Distance	Dentin substrate	Adhesive classification	µTBS Laser (MPa)	µTBS Control (MPa)	Aging	Outcome
Rezaee et al. (12)	810, 970	0.8	Continuous	20	16	1 mm	Coronal dentin	Universal adhesive	Slight increase (NS)	Comparable	24 h	No effect
Naik et al. (11)	970	1	Continuous	10	30	1 mm	Coronal dentin	Two-step etch-and-rinse adhesive	8.62 ± 0.49/9.25 ± 1.22	6.3 ± 1.17/7.13 ± 0.78	Immediate	Increase
Kasraei et al. (9)	940	1	Continuous	10	NR	1 mm	Coronal dentin	Two-step etch-and-rinse adhesive	38.35 ± 8.99	18.85 ± 4.79	Immediate	Increase
Zabeu et al. (10)	970	0.8	Pulsed (10 Hz)	30	24	Contact mode	Coronal dentin	Three-step etch-and-rinse/Two-step self-etch adhesive	No significant improvement	Comparable	Immediate + 12 months	No effect
Maenosono et al. (8)	970	1	Continuous	30	NR	Contact mode	Superficial dentin	Two-step etch-and-rinse/One-step self-etch adhesive	43.69 ± 8.15/29.87 ± 6.98	33.49 ± 6.77/19.67 ± 5.86	Immediate	Increase

### Risk of bias assessment

The methodological quality of the included studies was assessed using the QUIN (Quality Assessment Tool for *in vitro* Studies). This tool evaluates study quality across 12 predefined domains, including clarity of objectives, sample size justification, sample selection, randomization, control group presence, methodological detail, operator information, blinding, outcome measurement, statistical analysis, data presentation, and disclosure of conflicts of interest.

Each domain is scored on a three-point scale (0–2), where higher scores indicate better methodological reporting and lower risk of bias. Table 3 summarizes the scoring criteria used for each domain in the present review.

**Table 3:** Scoring criteria for risk of bias.

Domain	Criterion	Score 0	Score 1	Score 2
Aims	Clear statement of objectives	Not reported	Partially stated	Clearly stated
Sample size	Justification provided	Not reported	Mentioned but no calculation	Power/sample calculation reported
Sample Selection	Inclusion/exclusion criteria	Not reported	Partially described	Fully described
Randomization	Random allocation of specimens	Not reported	Implied but not described	Explicitly described
Control	Appropriate control group	Absent	Present but inadequate	Adequate control
Methodology	Reproducible protocol	Not reproducible	Partially described	Fully reproducible
Operator	Calibration/training	Not reported	Partially described	Fully described
Blinding	Assessor blinding	Not reported	Partial blinding	Full blinding
Outcome	Valid measurement method	Not valid	Partially valid	Validated method
Statistics	Appropriate analysis	Inappropriate	Partially appropriate	Fully appropriate
Data	Complete presentation	Incomplete	Partially complete	Complete
COI	Conflict disclosure	Not reported	Partial disclosure	Full disclosure

**Figure 2:**
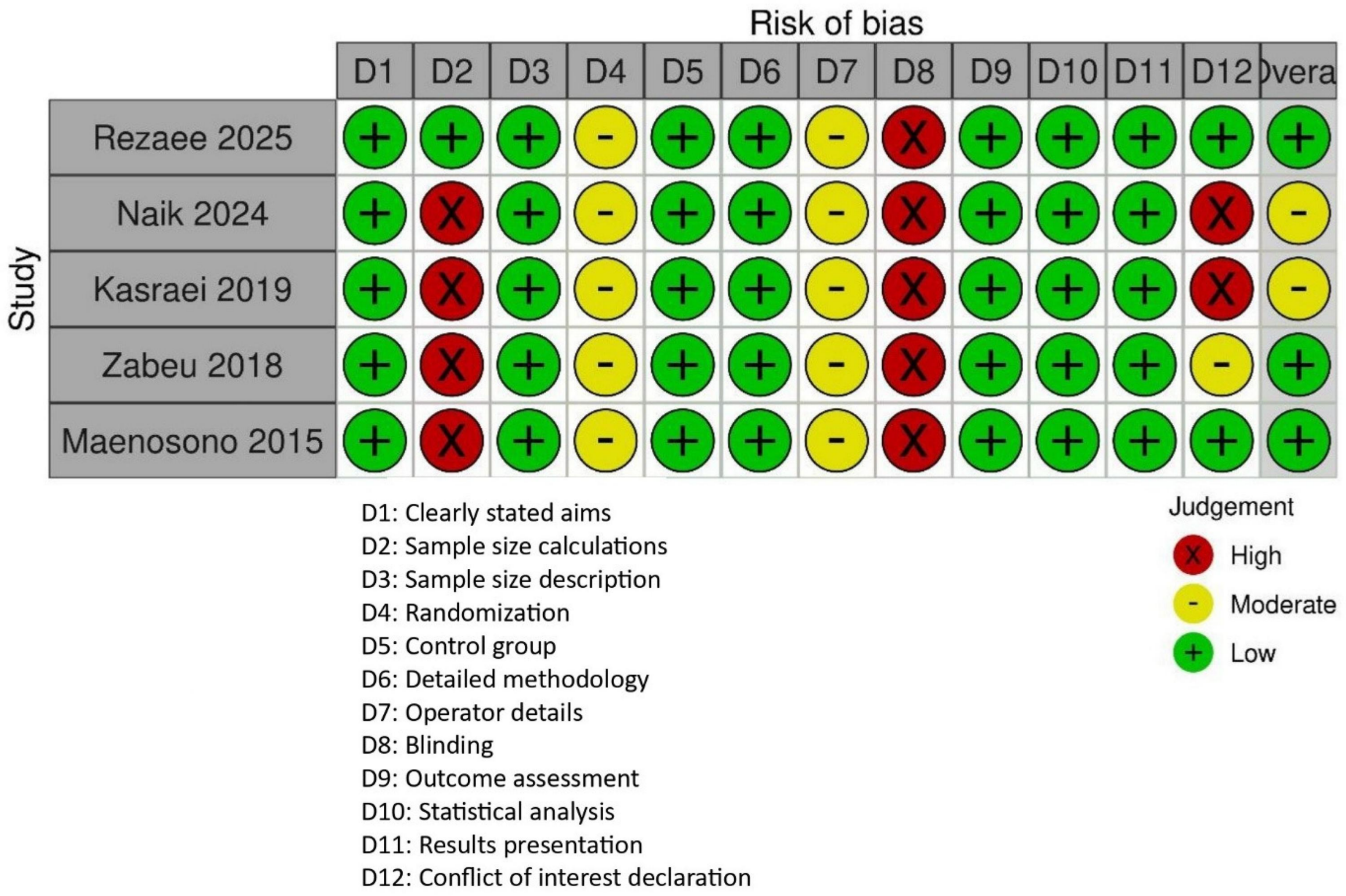
Risk of bias. Generated using the robvis tool, “Example risk of bias traffic light plot of ROB2 assessments created using robvis and the colourblind palette” by Luke A. McGuinness and Julian P. T. Higgins, licensed under CC BY 4.0.

The overall QUIN score for each study was calculated as a percentage using the formula: (total score obtained ÷ maximum possible score) × 100. Based on the criteria proposed in the original QUIN tool, studies scoring >70% were classified as having a low risk of bias, those scoring between 50% and 70% were classified as having a moderate risk of bias, and those scoring <50% were classified as having a high risk of bias (18). Higher scores indicate greater methodological rigor, more comprehensive reporting, and a lower likelihood of bias influencing the study findings. The domain-wise QUIN risk-of-bias assessment of the included studies is presented in Figure 2.

## Results

The literature search produced a total of 101 articles and after removing duplicates, 85 studies were screened, after which 23 articles underwent full-text screening. Finally, 5 articles were found eligible for the review. A customized data extraction template was employed to extract information from all the eligible studies, utilizing the following predefined headings.

### Study characteristics

Key study characteristics are summarized in Table 2 and are described below.

1. Rezaee et al. (12): This recent study evaluated the effects of 810 and 970 nm diode lasers using a 0.8 W power setting in continuous mode for 20 s (16 J total energy). The researchers applied these parameters to coronal dentin in conjunction with universal adhesives. They reported only a slight, non-significant increase in bond strength, ultimately concluding that the laser treatment had no significant effect on µTBS.
2. Naik et al. (11) evaluated the effect of 970 nm diode laser irradiation on the microtensile bond strength of an etch-and-rinse adhesive system using phosphoric acid and glycolic acid etchants. Laser-treated groups demonstrated significantly higher µTBS values than their corresponding control groups (PA-DL: 8.62 ± 0.49 vs. PA: 6.3 ± 1.17 MPa, *P* < 0.001; GA-DL: 9.25 ± 1.22 MPa vs. GA: 7.13 ± 0.78 MPa, *P* < 0.001). The highest bond strength was observed in the GA-DL group.
3. Kasraei et al. (9): Investigating a 940 nm wavelength, this study applied 1 W in continuous mode for 10 s. They focused on coronal dentin using a two-step etch-and-rinse adhesive (Single Bond 2). This study reported a substantial improvement, with µTBS nearly doubling from 18.85 MPa in the control to 38.35 MPa in the laser-treated group.
4. Zabeu et al. (10): This study was unique in using a 970 nm laser in pulsed mode (10 Hz) at 0.8 W for 30 s (24 J). They tested both three-step etch-and-rinse and two-step self-etch adhesives on coronal dentin. Notably, they also assessed long-term durability through 12 months of water storage, finding that while there was no significant improvement initially, bond strength decreased across all groups over time.
5. Maenosono et al. (8): Utilizing a 970 nm wavelength at 1 W in continuous mode for 30 s, this study targeted superficial dentin. They employed two-step etch-and-rinse and one-step self-etch adhesives. The results showed a statistically significant enhancement in µTBS for both adhesive types following laser irradiation.

### Risk of bias

The QUIN risk-of-bias assessment for the five included studies is summarized in Table 3. Based on the predefined QUIN scoring criteria, the total scores and classifications were as follows: Rezaee et al. (12) achieved a score of 20/24 (83.3%) and was classified as having a low risk of bias; Naik et al. (11) and Kasraei et al. (9) each achieved a score of 16/24 (66.7%) and were classified as having a moderate risk of bias; Zabeu et al. (10) achieved a score of 17/24 (70.8%) and was classified as having a low risk of bias; and Maenosono et al. (8) achieved a score of 18/24 (75.0%) and was also classified as having a low risk of bias. Overall, three studies (60%) were categorized as low risk of bias and two studies (40%) as moderate risk of bias, with no studies classified as high risk of bias. The detailed domain-wise QUIN scores and overall risk classifications for each included study are presented in Table 4.

**Table 4:** Risk of bias.

Domain	Rezaee et al. (12)	Naik et al. (11)	Kasraei et al. (9)	Zabeu et al. (10)	Maenosono et al. (8)
Clearly stated aims	2	2	2	2	2
Sample size calculation	2	0	0	0	0
Sample selection description	2	2	2	2	2
Randomization	1	1	1	1	1
Control group	2	2	2	2	2
Detailed methodology	2	2	2	2	2
Operator details	1	1	1	1	1
Blinding	0	0	0	0	0
Outcome assessment	2	2	2	2	2
Statistical analysis	2	2	2	2	2
Results presentation	2	2	2	2	2
Conflict of interest declaration	2	0	0	1	2
Total (/24)	20	16	16	17	18
Percentage (%)	83.3%	66.7%	66.7%	70.8%	75.0%
Risk category	Low	Moderate	Moderate	Low	Low

The domain-wise QUIN assessment indicated that all included studies clearly stated their research objectives and provided adequate descriptions of sample selection, experimental procedures, outcome assessment methods, and statistical analyses. Appropriate control groups were incorporated in all studies, and the reported methodologies were sufficiently detailed to permit reproducibility. Although specimen randomization was reported across all investigations, the specific methods used to generate the random allocation sequence were not described, resulting in partial scores for the randomization domain. Similarly, information regarding operator calibration, training, or experience was only partially reported. None of the included studies reported operator or assessor blinding, making this the most consistent methodological limitation identified. Furthermore, formal sample size calculations were reported in only one study, while the remaining studies did not provide a justification for sample size determination. Conflict-of-interest declarations were inconsistently reported across studies. Despite these limitations, the included studies generally demonstrated acceptable methodological quality, with validated outcome measures and appropriate analytical approaches being consistently employed.

### Result of individual studies

A total of five *in-vitro* studies evaluating the effect of diode laser-mediated dentin biomodification on microtensile bond strength were included in this review. All studies utilized human permanent dentin and diode lasers within the wavelength range of 810–970 nm. Although the review eligibility criteria encompassed diode laser wavelengths ranging from 780 to 980 nm, no eligible studies evaluating 780 or 980 nm diode lasers were identified. Consequently, the available evidence was limited to wavelengths between 810 and 970 nm. Despite variations in adhesive strategies, irradiation timing, and experimental protocols, three of the five studies demonstrated an increase in µTBS following diode laser application, while two studies reported no significant improvement.

Maenosono et al. reported a significant enhancement in bond strength when diode laser irradiation was applied after adhesive placement and prior to polymerization, attributing this effect to improved monomer infiltration and solvent evaporation. Similarly, Kasraei et al. demonstrated that 940 nm diode laser irradiation significantly increased bond strength, with the greatest improvement observed when irradiation was performed after adhesive application. In agreement with these findings, Naik et al. observed higher µTBS values in laser-treated groups compared to controls, irrespective of the etchant used, indicating that diode laser pretreatment can enhance bonding effectiveness across different etching protocols.

In contrast, both Zabeu et al. and Rezaee et al. reported no statistically significant improvement in bond strength following diode laser irradiation. Zabeu et al. further demonstrated a reduction in bond strength across all groups after long-term water storage, suggesting limited influence of laser treatment on bond durability. Similarly, Rezaee et al. observed only a slight increase in bond strength values in laser-treated groups, which was not statistically significant, indicating that the effectiveness of diode laser application may depend on specific irradiation parameters and adhesive protocols.

Percentage increases were calculated from reported mean µTBS values in Table 2 and the relative increase in µTBS in laser-treated groups ranged approximately from 30% to over 100%, although variability across adhesive systems and protocols was substantial.

## Discussion

This systematic review synthesized the available *in-vitro* evidence regarding the effect of diode laser-mediated dentin biomodification on microtensile bond strength (µTBS) at the resin-dentin interface. The overall findings suggest that diode laser pretreatment may improve resin–dentin bonding; however the extent of improvement is influenced by laser parameters, adhesive strategy, and experimental conditions (8–12).

Three of the five included studies demonstrated an increase in µTBS following diode laser treatment. The mechanism underlying this enhancement may be attributed to thermally induced collagen modification, reduction in dentinal fluid movement, and alterations in the smear layer that facilitate improved resin infiltration. Diode lasers, operating primarily through photothermal interactions, can denature or compact collagen fibrils, creating a more receptive substrate for monomer diffusion (5, 6). These findings are consistent with previous reports indicating that laser-induced biomodification may stabilize collagen matrices, reduce enzymatic degradation, and improve hybrid layer formation and durability.

The variability in outcomes across the included studies can also be associated with differences in adhesive systems. The included studies employed a range of adhesive systems including etch-and-rinse (Adper Single Bond 2; Single Bond 2), self-etch (Clearfil SE), multi-mode (Scotchbond MP), and universal adhesives (Three-N-Bond, G-Premio Bond, Single Bond Universal). Notably, the study employing universal adhesives (12) found no statistically significant improvement following laser pretreatment, suggesting that the presence of functional monomers alone does not guarantee a laser-enhanced bonding benefit. The study employing Etch-and-rinse systems (8, 9) demonstrated the most pronounced improvements, possibly because post-laser dentin surface energy increases may more effectively support the hydrophilic etching and rinsing mechanism.

However, the results were not consistent across all studies. Both Zabeu et al. and Rezaee et al. reported no statistically significant improvement in bond strength following diode laser irradiation (10, 12). Zabeu et al. further demonstrated a reduction in bond strength after long-term water storage, indicating limited influence of laser treatment on bond durability (10). Similarly, Rezaee et al. observed only a slight, non-significant increase in bond strength despite using diode lasers at 810 and 970 nm wavelengths (12). These findings highlight that the effectiveness of diode laser pretreatment is not universal and may depend on specific irradiation parameters, adhesive systems, and application protocols.

Laser parameters, including wavelength, power output, energy density, and irradiation duration, play a critical role in determining treatment outcomes. Excessive thermal energy may lead to adverse effects such as collagen degradation, carbonization, or microcrack formation, whereas insufficient energy may fail to induce meaningful structural changes (5, 6). The lack of standardized irradiation protocols across studies remains a significant limitation and contributes to heterogeneity in reported outcomes.

Ageing procedures, when assessed, demonstrated bond strength reduction across all experimental groups after 12 months of water storage (10), with no statistically significant difference between laser-treated and control groups at the aged time point. This finding suggests that diode laser pretreatment as applied in that study did not meaningfully protect the resin-dentin interface against long-term hydrolytic degradation. This finding is clinically relevant, as degradation of the resin-dentin interface over time is a primary factor contributing to restoration failure (2, 3).

The findings of this review are contextualised within the broader landscape of dentin biomodification strategies. Proanthocyanidin-based collagen cross-linking has been shown to significantly increase µTBS and inhibit MMP-mediated hybrid layer degradation in multiple *in vitro* studies, with mean improvements of 20%–40% over untreated controls (13). Similarly, carbodiimide (EDC) application prior to adhesive bonding has demonstrated statistically significant improvements in both immediate and aged µTBS (14). Riboflavin/UVA cross-linking, while promising for collagen stabilisation, requires additional irradiation equipment and has shown variable bonding outcomes (15). Compared with these chemical biomodification strategies, diode laser pretreatment offers procedural simplicity and reduced chair-time without chemical application, but currently demonstrates less consistent evidence of bonding improvement. Future comparative *in vitro* trials directly pitting diode laser against proanthocyanidin or EDC pretreatment under standardised conditions would substantially advance the clinical translation of these findings.

However, several limitations must be acknowledged. All included studies were laboratory-based, which restricts direct extrapolation to clinical conditions. Although specimen randomization was reported across the included studies, the methods used to generate the random allocation sequence were not clearly described. Furthermore, none of the studies reported operator or assessor blinding, introducing a potential risk of bias. Variations in specimen preparation, adhesive protocols, and µTBS testing methodology further contribute to heterogeneity among the included studies (7).

Despite these limitations, the overall body of evidence supports the potential utility of diode laser pretreatment as an adjunctive strategy for improving dentin bonding. Standardized irradiation protocols, optimized laser parameter settings, and well-designed translational studies are required to confirm the clinical relevance of diode laser-mediated dentin biomodification.

The limited number of eligible studies (*n* = 5) reflects both the specificity of the research question and the stringent inclusion criteria applied. While these criteria enhance methodological consistency, they also underscore the need for further well-designed studies in this area. Although diode laser pretreatment demonstrates promising laboratory outcomes, clinical extrapolation should be approached cautiously. At present, diode laser-mediated dentin biomodification should be considered an adjunctive and experimental approach until validated through controlled clinical trials.

### Impact of bias on study results

The methodological limitations identified through the QUIN assessment should be considered when interpreting the findings of this review. In particular, the absence of operator or assessor blinding across all included studies may have introduced measurement bias during specimen preparation, testing, or outcome evaluation. Similarly, although specimen randomization was reported, the lack of detailed descriptions of randomization procedures may have increased the risk of selection bias. In addition, the absence of formal sample size calculations in most studies raises the possibility of inadequate statistical power, potentially affecting the reliability and precision of the reported outcomes. These sources of bias may have contributed to variability in the observed microtensile bond strength values and could have influenced the magnitude of the reported treatment effects. Therefore, while the overall findings suggest a potential benefit of diode laser pretreatment under specific irradiation conditions, the results should be interpreted with caution until confirmed by methodologically rigorous studies with standardized protocols and comprehensive reporting.

### Implications for future research

Future studies should assess interactions with modern universal adhesive systems and concentrate on standardised diode laser irradiation techniques. Furthermore, to confirm the translational relevance of laser-mediated dentin biomodification, long-term ageing studies and carefully planned clinical trials are required.

## Limitations

The findings of this review should be interpreted in light of certain limitations. First, the number of eligible studies included was limited, which restricts the strength of the evidence and the ability to draw definitive conclusions. In addition, there was considerable heterogeneity among the included studies with respect to laser parameters, such as wavelength, power settings, and irradiation time, as well as differences in the adhesive systems used. Variations in microtensile bond strength (µTBS) testing protocols, including specimen preparation, cross-head speed, and aging methods, further contributed to methodological inconsistency and limited direct comparison of outcomes. Moreover, the predominantly *in-vitro* nature of the included studies may not accurately replicate the complex oral environment, thereby limiting the extrapolation of these results to clinical practice. In addition, only studies published in the English language were included. Consequently, relevant studies published in other languages may have been missed, introducing the potential for language bias. The substantial clinical and methodological heterogeneity among the included studies precluded quantitative meta-analysis and limited direct comparison of outcomes.

## Conclusion

Based on the synthesis of evidence from five *in-vitro* studies, diode laser-mediated dentin biomodification suggests a potential beneficial effect on µTBS under optimised irradiation conditions, though limited by study heterogeneity. The observed improvements in resin-dentin bonding are likely attributable to laser-induced modifications of the dentin substrate, including collagen fibril alteration, smear layer modification, and enhanced resin infiltration, which collectively contribute to improved hybrid layer quality.

However, the magnitude and consistency of the bonding improvement are strongly influenced by laser wavelength, power output, irradiation duration, and the type of adhesive system used. Variability in experimental design, incomplete reporting of laser parameters, and differences in µTBS testing methodologies limit direct comparison across studies and preclude definitive conclusions regarding optimal clinical protocols.

Given that all included investigations were laboratory-based, the findings should be interpreted cautiously, as *in-vitro* conditions cannot fully replicate the complex biological and mechanical challenges present in the oral environment. Consequently, while diode laser pretreatment appears to be a promising adjunctive strategy for dentin bonding, its routine clinical application cannot yet be recommended.

Future research should focus on well-designed, standardized *in-vitro* studies with comprehensive reporting of laser parameters, followed by controlled *in-vivo* and long-term clinical trials. Such investigations are essential to identify evidence-based irradiation thresholds, evaluate long-term bond durability under simulated oral ageing conditions, and ultimately establish whether diode laser pretreatment constitutes a clinically viable and reproducible adjunct to contemporary adhesive bonding protocols.

## Data Availability

The original contributions presented in the study are included in the article/Supplementary Material, further inquiries can be directed to the corresponding author.
